# Climatic Factors Driving Invasion of the Tiger Mosquito (*Aedes albopictus*) into New Areas of Trentino, Northern Italy

**DOI:** 10.1371/journal.pone.0014800

**Published:** 2011-04-15

**Authors:** David Roiz, Markus Neteler, Cristina Castellani, Daniele Arnoldi, Annapaola Rizzoli

**Affiliations:** 1 Department of Biodiversity and Molecular Ecology, Fondazione Edmund Mach, Research and Innovation Centre, S. Michele all' Adige, Italy; 2 Wetland Ecology Department, Doñana Biological Station (CSIC), Seville, Spain; University of Liverpool, United Kingdom

## Abstract

**Background:**

The tiger mosquito (*Aedes albopictus*), vector of several emerging diseases, is expanding into more northerly latitudes as well as into higher altitudes in northern Italy. Changes in the pattern of distribution of the tiger mosquito may affect the potential spread of infectious diseases transmitted by this species in Europe. Therefore, predicting suitable areas of future establishment and spread is essential for planning early prevention and control strategies.

**Methodology/Principal Findings:**

To identify the areas currently most suitable for the occurrence of the tiger mosquito in the Province of Trento, we combined field entomological observations with analyses of satellite temperature data (MODIS Land Surface Temperature: LST) and human population data. We determine threshold conditions for the survival of overwintering eggs and for adult survival using both January mean temperatures and annual mean temperatures. We show that the 0°C LST threshold for January mean temperatures and the 11°C threshold for annual mean temperatures provide the best predictors for identifying the areas that could potentially support populations of this mosquito. In fact, human population density and distance to human settlements appear to be less important variables affecting mosquito distribution in this area. Finally, we evaluated the future establishment and spread of this species in relation to predicted climate warming by considering the A2 scenario for 2050 statistically downscaled at regional level in which winter and annual temperatures increase by 1.5 and 1°C, respectively.

**Conclusions/Significance:**

MODIS satellite LST data are useful for accurately predicting potential areas of tiger mosquito distribution and for revealing the range limits of this species in mountainous areas, predictions which could be extended to an European scale. We show that the observed trend of increasing temperatures due to climate change could facilitate further invasion of *Ae. albopictus* into new areas.

## Introduction

The Asian tiger mosquito, *Aedes albopictus* (Skuse, 1894) (Diptera: Culicidae), is native to the forests of south-east Asia, where it breeds in tree-holes. Although it does not fly further than half a kilometre [1], over the past 30 years this invasive species has been introduced to the American, Indo-Pacific and Australian regions, as well as to Europe and Africa, by transportation of eggs (which are drought-resistant for several months), mainly in used tires or Lucky Bamboo plants (*Dracaena sp*.) [2].The first record of *Ae. albopictus* in Italy was in Genoa in the late summer of 1990 [3], while the first established populations were identified in Padua (Region of Veneto) in 1991, probably introduced from the United States in used tires [4]. The tiger mosquito has subsequently spread throughout the peninsula, with populations now established in almost all regions of Italy [2]. In the Province of Trento, this species was first recorded in 1996 in a used tire depot near Rovereto (30 km south of Trento) [5] and has now been recorded throughout the municipalities of Rovereto, Arco and Riva del Garda [6].

In addition to being a nuisance, the biting insect *Ae. albopictus* is an efficient laboratory vector of at least 22 arboviruses [7], including dengue (DEN) and Chikungunya (CHIK) viruses [8], [9]. Experiments have shown that European populations are able to replicate and transmit CHIK and DEN viruses at high levels of viral replication and can even transmit the CHIK virus at day 2 after infection [10]. This risk was recently demonstrated by the CHIK outbreak in Italy in 2007 in the Region of Emilia-Romagna [11] and in the cases of Dengue virus (DEN) in France [12]. In addition to CHIK and DEN, several other pathogens have been detected in field populations of *Ae. albopictus:* West Nile virus [13], [14], eastern equine encephalitis, yellow fever, La Crosse, Japanese encephalitis, Potosi, Jamestone Canyon, Tensaw, Keystone, *Dirofilaria immitis* and *D. repens* [7].

*Aedes albopictus* is considered an ecological generalist, and has apparently been able to adapt to both tropical and temperate climates. Temperate populations of *Ae. albopictus* are able to produce diapausing eggs, allowing the species to survive the winter period [15], and have adapted to reproducing in a wide range of containers manufactured by humans [1]. This species mainly colonizes urban and suburban areas, where female mosquitoes frequently use humans as hosts for bloodmeals.

Previous publications indicate that the distribution of *Ae. albopictus* is determined by several environmental variables [16], such as winter and summer temperatures, precipitation patterns and photoperiod. January mean temperatures (JanT^mean^) affect the survival rate of diapausing eggs during the winter period [1] since a low JanT^mean^ leads to significant egg mortality. An air temperature of 0°C is the generally accepted threshold for JanT^mean^ [8], [17]. Annual mean temperatures (AnnT^mean^) determine the areas suitable for adult survival, with 11°C as the generally accepted AnnT^mean^ threshold value [18]. Apart from temperature, annual precipitation is another important ecological indicator of the areas where mosquito populations may thrive as it conditions the maintenance of larval habitats. 500 mm is the significant threshold value [8], [19], [20], but the species is also found in areas with lower precipitation [21]. In areas with high precipitation, on the other hand, host-seeking female abundance was negatively correlated with accumulated precipitation [6]. Regional differences in precipitation affect the distribution of *Ae. albopictus* in the U.S.A. [16], while precipitation patterns have been proposed as a limiting factor in Mediterranean areas, due to the seasonality of precipitation, with a suggested minimum threshold of 60 days of precipitation [20]. In our study area, annual rainfall is around 1,100 mm and precipitation is therefore not considered a limiting factor. Other ecological and human factors are also relevant for the distribution of *Ae. albopictus* populations, such as land use/land cover, type of urbanization and human population density, all of which may influence both distribution and density [22]. Some authors consider altitude to be an important limiting factor in the distribution of *Ae. albopictus*, which has been detected at altitudes of up to 600 m in Italy [2].

Geographic Information Systems (GIS) and Remote Sensing complement studies on *Ae. albopictus* by providing maps of potential establishment areas [17], [23]–[27]. Predicted global climate change is likely to extend the northern distribution of *Ae. albopictus* and to place further limitations on its establishment in arid regions [16]. Changes in the pattern of distribution of the tiger mosquito will affect the potential spread of infectious diseases transmitted by this species in Europe, and are hence of particular concern. Forecasting these changes is an important factor in preventing further spread into susceptible areas.

The aim of this study is to determine the most suitable distribution areas of *Ae. albopictus* in the Province of Trento, northeastern Italy, by comparing field entomological data with satellite temperature data (MODIS LST) and indices of human population density. In addition, we attempted to forecast the effect of the expected increase in temperature on the suitable distribution areas on the basis of the most reasonable predictions obtained after downscaling the A2 scenario for 2050 to a regional level.

## Materials and Methods

The study was carried out in northeastern Italy in Trentino, the Autonomous Province of Trento (Region of Trentino-Alto Adige). The Province of Trento (latitude limits: 46.5332N (north), 45.6730N (south); longitude limits: 10.4522E (west), 11.9632E (east)), is located in a mountainous region on the southern side of the Alps and covers an area of 6,200 km^2^. With 519,000 inhabitants, it has a low human population density compared to other Italian regions. In general, the climate can be considered temperate-oceanic with four main subclimatic areas: sub-Mediterranean (close to Lake Garda, leading to mild winters), sub-continental (the main river valleys, having more severe winters), continental (the alpine valleys) and alpine (the areas above the tree line). More than 70% of the territory lies over 1,000 m above sea level, and about 55% is covered by coniferous and deciduous forests. The region has a wide variety of habitats which support Mediterranean tree species, such as *Quercus ilex* and *Olea europae*, subalpine species such as *Pinus* and *Picea*, as well as alpine species and mountain grasses.

In the sub-Mediterranean area located at the northern end of Lake Garda, the climate, human population density and subsequent presence of artificial containers typical of modern urbanization (free-standing houses, chalets) not only provide an excellent habitat for the occurrence of autochthonous species such as *Culex pipiens*, but also for the invasive tiger mosquito *Ae. albopictus.*

In order to assess current *Ae. albopictus* distributional patterns in the Trento Province, we investigated the mosquito's presence throughout areas of potential habitat. Oviposition traps (ovitraps) were used as a standard tool for monitoring the incidence of container-inhabiting mosquitoes, such as *Ae. aegypti* and *Ae. albopictus*. However, studies have shown that ovitraps measure the occurrence but not the abundance of adults, and that collecting eggs in ovitraps is more accurate than collecting adults for detecting *Aedes,* especially at low population levels [28]. Therefore, in our study, we made some modifications to the standard methodologies [29]. In designing the present study we took into account the fact that *Ae. albopictus* has a maximum flight range of 500 m [1], [30], but can be displaced over much longer distances via passive transport along the roadway system [31]. Therefore, we positioned 145 sample stations along the roads running north and of previously documented distribution ‘hot spots’ (Rovereto, Arco and the Riva del Garda area) at distances of more than 500 m apart, and also included a comprehensive survey of the city of Trento. Each sample station consisted of one ovitrap placed in a sheltered site shaded by vegetation. The locations of all traps were recorded by GPS (GeoExplorer 2005 series, Trimble). Ovitraps were checked fortnightly from June to November for the presence of mosquito eggs. The water in the ovitrap was frequently checked for hatched mosquito larvae and/or pupae, and Bti: *Bacilus thuringhiensis* var. *israeliensis* (VectoBac, ValentBioSciences) granules were added to it to prevent larval development. The number of eggs collected per trap was assessed by examination under a stereomicroscope. Since the eggs could belong to other tree-hole dwelling *Aedes* and *Ochlerotatus* (*Ae. geniculatus*, *Ae. echinus*, *Ae. berlandi*) [32], samples were kept in moist conditions for five days and then flooded in the same plastic drawers used for WFC collection. Resulting larvae were stored in 70% alcohol and identified using the key of Schaffner et al. [33]. When *Ae. albopictus* was confirmed as both eggs and larva, the ovitraps were removed, as the aim was to detect species presence but not to evaluate density.

In this study, we used data from the Terra and Aqua satellites. Both carry the MODIS (Moderate Resolution Imaging Spectroradiometer) sensor and together provide four global coverages per day at various pixel resolutions. Especially relevant are the daily Land Surface Temperature (LST) maps (originally 1000 m pixel resolution; available from https://wist.echo.nasa.gov) which allow temperature-based indicators to be derived in a GIS framework. To spatially match the LST maps to the existing GIS data, we reprojected them from the original Sinusoidal projection to the UTM32 cartographic system using the MODIS Reprojection Tool (MRT, version 4.0 from U.S. Geological Survey). In this step, the resolution was increased to 200 m pixels and the values converted from Kelvin to degrees Celsius [34], [35]. Since the original LST maps can be cloud-contaminated or have missing pixels due to other problems, we reconstructed all maps to complete maps before using them for our study [35]. To do this, we processed more than 11,000 daily MODIS LST scenes from the study area from 3/2000 to 2/2009 in a GIS framework (GRASS GIS 6.4, GRASS Development Team 2009, http://grass.osgeo.org). This reconstruction of the daily LST maps was done by filtering all clouds and poor quality pixels and subsequently filling the resulting no-data areas in the maps with a temperature-gradient-based model [35]. From the final, completed LST map set, we prepared the required temperature indicator maps by aggregating minimum and maximum temperatures to obtain JanT^mean^ and AnnT^mean^. In order to create the MODIS LST JanT^mean^ and AnnT^mean^ maps, we integrated all the pixels for the period 2001–09 with a JanT^mean^ above a chosen threshold of 0°C and with an AnnT^mean^ above a chosen threshold of 11°C, these being the thresholds which best fitted our field records of *Ae. albopictus*. For the final map, the areas where both indicators overlapped were plotted for the period 2001–2009 and integrated with three categories: 1) Highly suitable: this area includes all pixels with both indicators (JanT^mean^ and AnnT^mean^) above their thresholds of 0°C and 11°C respectively; 2) Moderately suitable: this area includes all the pixels where only one of the two indicators is above its threshold; and 3) Unsuitable: all the areas where neither of the two indicators is above the threshold.

For the statistical analysis, mean data from the years 2001–09 of the MODIS LST JanT^mean^ and AnnT^mean^ maps were extracted from the reconstructed database for each trap. We calculated distances between human population centers within the potential distribution area of *Ae. albopictus* and all ovitraps in the study area. Human population data was based on the official population census of 2001 (ISTAT, http://www.istat.it) and from Landscan Global Population Database (http://www.ornl.gov/landscan/). The human population variable was log-transformed to reduce the influence of outliers and bring the data closer to a normal distribution.

To investigate the effect of climate and human population variables on *Ae. albopictus* egg presence, we performed model building with multiple logistic regressions (Generalized linear model with binomial distribution and logit link). The response variable, presence/absence of *Ae. albopictus*, was examined in relation to AnnT^mean^ LST, JanT^mean^ LST, human population (log transformed) and distance to human population centers. The output of the best model is presented and discussed. In addition, we used the Akaike information criterion and normalized Akaike weights [36] to assess the probability that a specific hypothesis was the most likely of those considered. We also used the sum of Akaike weights across models containing a specific variable (e.g. JanT^mean^) to assess the importance of specific variables in explaining variation in *Ae. albopictus* presence.

All statistical analyses were performed using STATISTICA version 8.0 (StatSoft, Tulsa, USA) and R version 2.10.1 [37] and results were considered significant if P<0.05.

Finally, we generated a scenario based on the SRES A2 scenario for the decade 2040–2050 in order to evaluate the possible expansion of areas currently populated by *Ae. albopictus* as a result of the predicted increase in temperatures. With respect to the reference period 1961–90, an increase of 1.5°C in JanT^mean^ (Eccel et al., pers. comm.) and of 1°C in AnnT^mean^ [38] is predicted for 2050. We plotted the simulated JanT^mean^ and AnnT^mean^ for the period 2040–50 and integrated them in a final map with 3 categories as for the integration for the period 2001–09.

## Results

**Figure 1:**
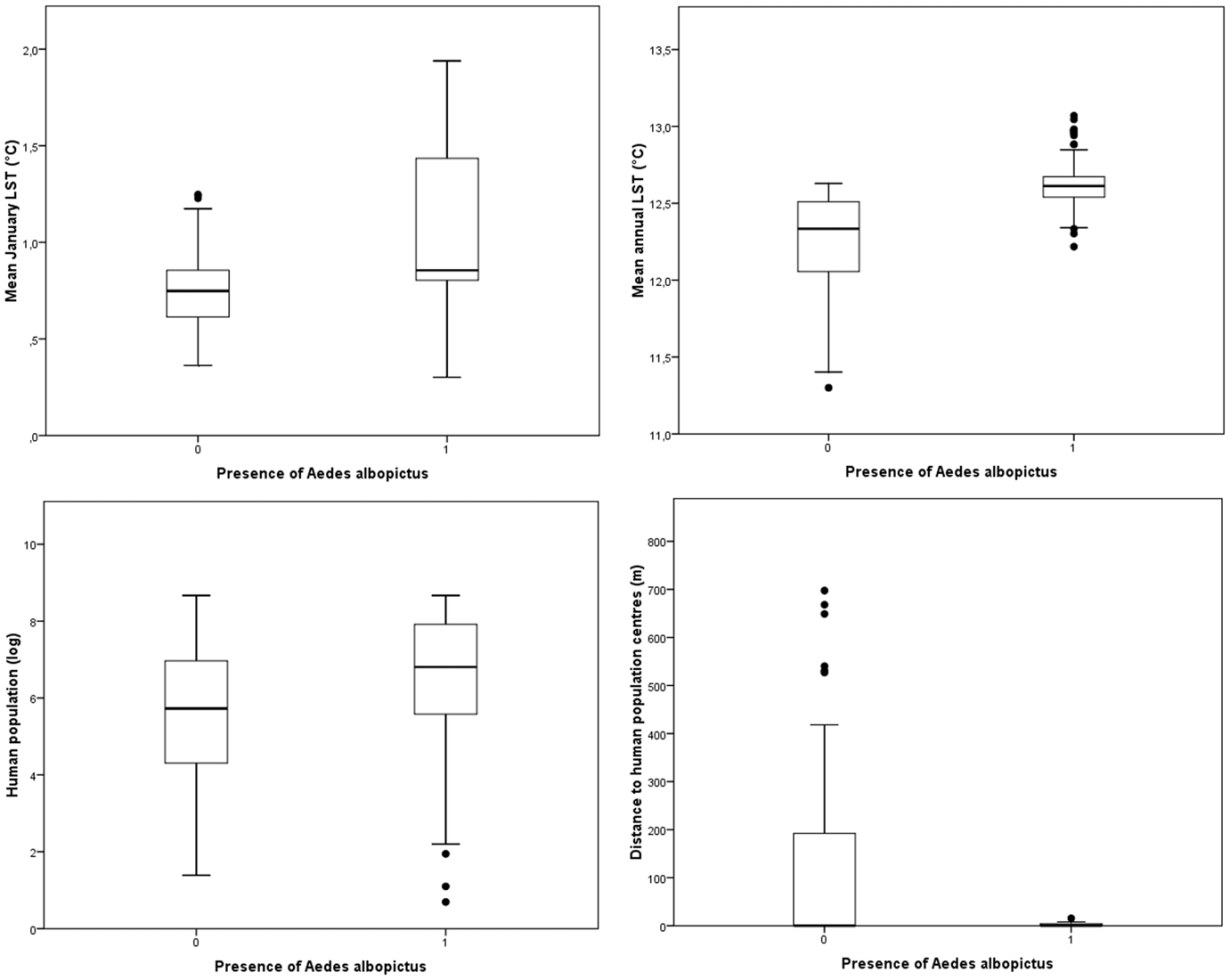
Differences between the areas with/without *Ae. albopictus* presence and the four explanatory variables: JanT^mean^ LST (top left), AnnT^mean^ LST (top right), human population density (log) (bottom left) and distance to human population centers (bottom right).

The influence of Land Surface Temperature variables (AnnT^mean^ and JanT^mean^) and human population indices (logarithm of human population density and distance to human population centers) was evaluated with a binomial generalized linear model (GLM) as a logistic regression. As shown in Table 1, different models were built and the best model was selected on the basis of AIC (Akaike Information Criterion), ΔAIC and Akaike weights [36]. Given that the top three models fall within 2 ΔAIC and include the four variables, we summed the Akaike weights of these variables in order to quantify the importance of each variable [36]. JanT^mean^ was the most important variable (sum of Akaike weight 0.9932) having a positive effect on mosquito incidence (Tables 1 and 2, Fig. 1 top left). AnnT^mean^ was the second most important variable (sum of Akaike weights 0.8627), also having a positive effect (Tables 1 and 2, Fig. 1 top right). Human population density (sum of Akaike weight 0.3798) and distance to human population centers (sum of Akaike weight 0.3262) were less important factors affecting the presence/absence of *Ae. albopictus* in the area (Table 1, Fig. 1 bottom left and right, respectively). Consequently, temperature variables were more crucial than human population variables for modeling *Ae. albopictus* potential distribution areas in this mountainous region.

**Table 1:** Results of the model building for multiple logistic regressions (Generalised linear models with binomial distribution and logit link).

Model	AIC	ΔAIC	Akaike weights
AnnTmean + JanTmean	206.47	0.00	0.4200
AnnTmean + JanTmean + distance	208.02	1.55	0.1935
AnnTmean + JanTmean + logpop	208.41	1.94	0.1592
JanTmean + logpop	209.70	3.23	0.0835
AnnTmean + JanTmean + logpop + distance	209.71	3.24	0.0831
JanTmean + logpop + distance	210.99	4.52	0.0438
JanTmean	214.80	8.33	0.0065
JanTmean + distance	216.02	9.55	0.0035
AnnTmean + logpop	216.65	10.18	0.0026
AnnTmean	217.10	10.63	0.0021
AnnTmean + distance	218.17	11.70	0.0012
AnnTmean + logpop + distance	218.53	12.06	0.0010
logpop	246.79	40.32	0.0000
logpop + distance	248.78	42.31	0.0000
distance	251.61	45.14	0.0000

Deviance and the Pearson Chi^2^ were not significant in all cases indicating no evidence for lack of fit of the model. Akaike weights were calculated based on [36].

**Table 2:** Output of the minimal adequate model of the Generalized Linear Model (GLM) with Binomial Distribution on *Ae. albopictus* presence (deviance: 200.47; Log-likelihood: -100.237).

Response variable	Explanatory variables	Coefficient (±S.E.)	Waldtest	d.f.	Pvalue
Presence of *Ae. albopictus*	January mean Land Surface Temperatures (JanT^mean^ LST)	2.5830±0.835	9.79	1	0.00175
	Annual mean Land Surface Temperature (AnnT^mean^ LST)	1.9623±0.654	8.993	1	0.00270

**Figure 2:**
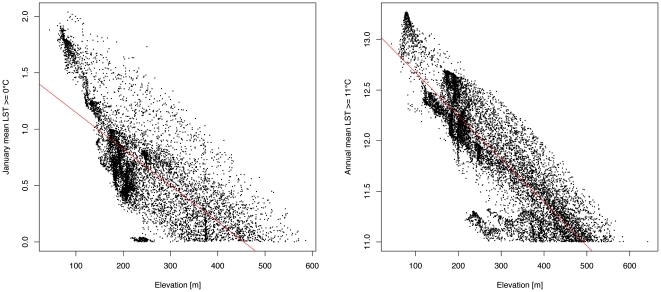
Relationship between the JanT^mean^ LST (left) and the AnnT^mean^ LST (right) and elevation. All the pixels with a JanT^mean^ LST > = 0°C and with an AnnT^mean^ LST > = 11°C were included and compared with elevation.

The relevance of altitude was studied in relation to the temperature thresholds of mean January LST (Fig. 2, left) and mean annual LST (Fig. 2, right). Overall, we did not observe a strong relationship between altitude and JanT^mean^ (Adjusted R^2^ = 0.527, p<0.001). However, a closer look revealed that the satellite-derived JanT^mean^ values have a high explanatory value, especially at low altitudes (Fig. 2, left). In this case, micro-climatic effects which could not be easily obtained from the meteorological station network are captured by the satellite data. A similar pattern, but with a higher correlation, was observed for the AnnT^mean^ (Adjusted R^2^ = 0.7472, p<0.001) (Fig. 2, right). In determining potential areas of *Ae. albopictus* distribution, satellite-observed Land Surface Temperatures deliver a more detailed picture than that obtained by considering only altitude as a variable. Detection of *Ae. albopictus* at 525 m around Pregasina near Riva del Garda (unpublished observations), where optimal temperatures are above the thresholds and altitude is relatively high for this species, supports this result.

**Figure 3:**
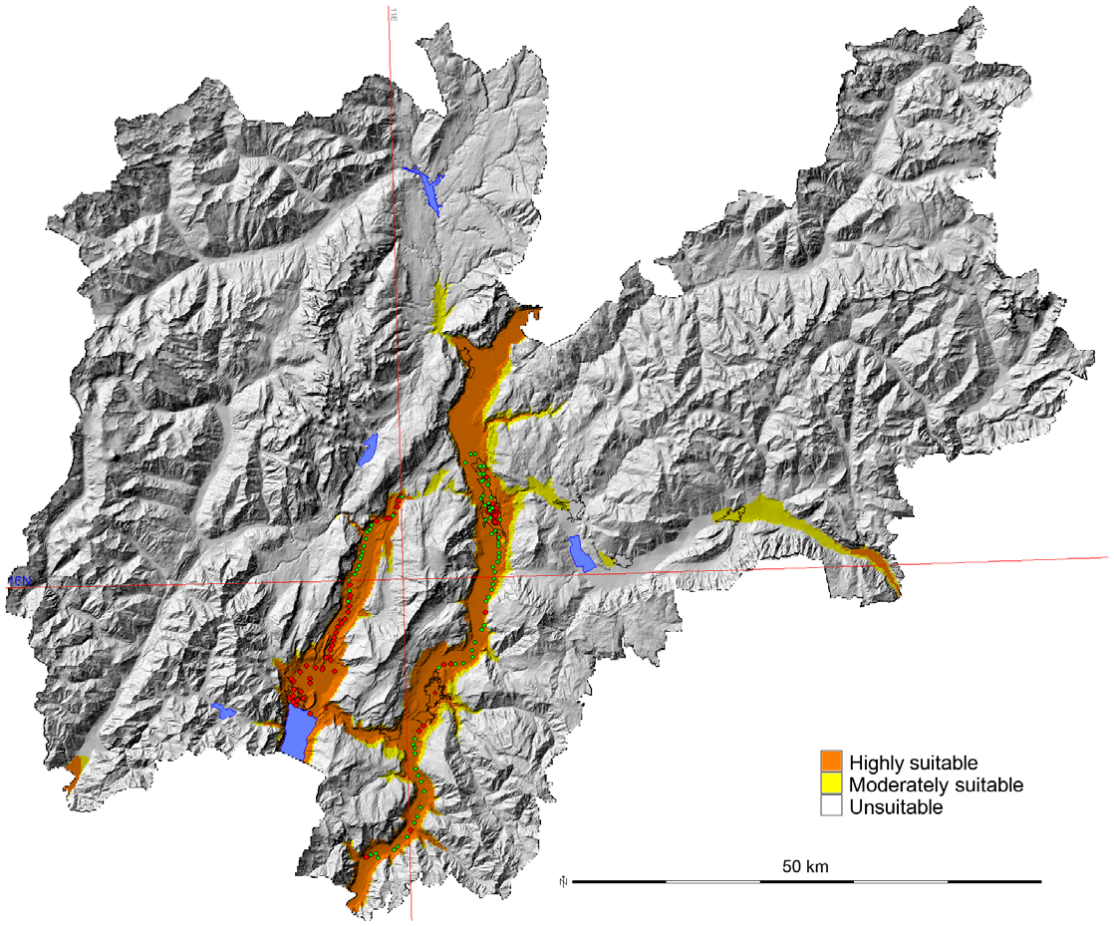
Potential and current distributional areas of *Ae. albopictus.* Overlap of both indicators (JanT^mean^ LST> = 0°C and AnnT^mean^ LST > = 11°C) were plotted for the period 2001–09 and integrated in a final map with 3 categories (see methods). Red spots represent the presence and green spots the absence of *Ae. albopictus.*

Figure 3 (red circles) represents the known distribution of *Ae. albopictus* in the study area in 2009. The species is widely distributed throughout Rovereto, Arco and Riva del Garda and is in an advanced stage of colonization. In our field work, new foci of this species have been detected to the north of Arco and Riva del Garda (in the Sarca Valley), with two isolated foci in the northern part of the Adige Valley and in the city of Trento, where this species was detected for the first time in 2008. In 2009, a comprehensive survey was carried out in Trento which tracked the expansion of this species across the city. Temperatures were especially low during the winter of 2008–2009, with a peak minimum temperature of −10°C recorded in the city and an air JanT^mean^ of −5°C.

During the study, several field stations in the valleys did not record any presence of this species (Fig. 3; green spots). However, some ovitraps located far from human dwellings were nonetheless positive for the tiger mosquito: in a natural area (Biotopo di Marco), in a parking lot for climbers (Pietramurata), at a bus stop (Lake Toblino) and in a parking lot (Mattarello).

**Figure 4:**
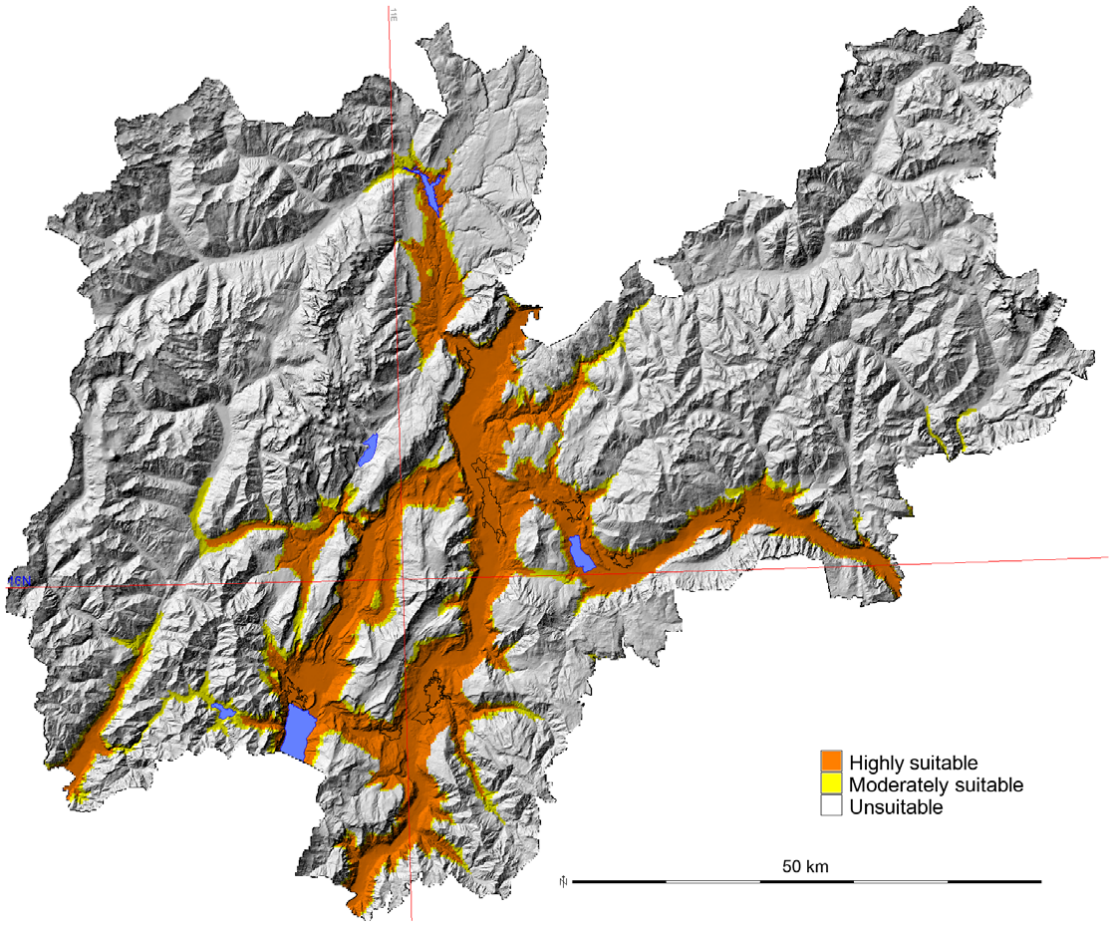
Potential distribution *of Ae. albopictus* in an A2 scenario for 2050 (see text). Overlap of both indicators (JanT^mean^ LST +1.5°C and AnnT^mean^ LST +1°C) were plotted for the study period and integrated in a final map with 3 categories (see methods).

Based on numerous previous studies [8], [16]–[20], [23], [25], [26], we built the optimal distribution model based on JanT^mean^ LST > = 0°C and AnnT^mean^ LST > = 11°C (Fig. 3). This model was intended to detect the optimal areas for *Ae. albopictus* in a mountainous area and fits with the current distribution. All the positive data fit inside the highly suitable areas. Finally, we constructed a scenario to forecast the expansion of *Ae. albopictus* population areas as a result of the predicted increase in temperatures for this area. In the scenario of climate change for 2050 (Fig. 4), the potential distribution area in the Province increases, especially in the central valleys, expanding eastwards through the Valsugana Valley and through the western valleys (Val di Sole, Sarche Valleys).

These results represent (to the best of our knowledge) the first evaluation at high-resolution to be made of the potential distribution areas of *Ae. albopictus*.

## Discussion

In recent years, the spread of *Ae. albopictus* throughout Europe has raised concern for future outbreaks of mosquito-borne diseases such as CHIK, in particular in light of the 2007 Italian outbreak. Therefore, the risk of future CHIK and DEN cases in areas where the tiger mosquito is spreading should be given serious consideration. It has been shown by several recent studies that modeling potential distribution areas for this invasive species is a valuable tool for preventing mosquito-borne diseases [17], [24], [26]. Importantly, these models need to be validated with empirical observations on a regional scale to assess and confirm the accuracy of their predictions. However, apart from the work by F. Schaffner et al. [26], most of the studies used environmental indicators based on air temperature threshold values (T_air_) are using data from Asian populations of this species [18]. We believe it is more relevant to base European thresholds on LST indicators, which allow the use of remote sensing techniques with MODIS data to be applied on a regional scale [35]. In this way, such data will be much more useful for health agencies, research bodies and authorities in developing an early warning system to prevent the spread of this invasive vector at the preliminary phase of colonization when control measures are highly effective.

Our present study makes use of two indicators based on LSTs validated on a regional scale with field data from the Province of Trento, northern Italy. This mountainous area of the Italian Alps (the Dolomites), where the tiger mosquito has been spreading since 1996 [5], was adopted as a model area for the northern distributional limit of this species in Italy [2]. Our aim was to develop models applicable to other mountain ranges of Europe where this species has not yet arrived and established itself (e.g., the Pyrenees, the Alps and other mountain ranges in France or Spain).We assumed temperature to be the most important limiting factor in our study area and in all areas where rainfall is not a limiting factor (mean annual precipitation above 500 mm), which includes most of Europe except parts of the Iberian peninsula, southern Italy, and some Italian islands, southern Greece, Turkey and Bulgaria [8], [19]. Our work is therefore based on the assumption that these two temperature indicators – mean January LST (JanT^mean^) and mean annual LST (AnnT^mean^) – are the crucial variables that limit the distribution of *Ae. albopictus* in the area under investigation. Other human and ecological factors are also relevant for determining the distribution of *Ae. albopictus* populations [22]. We showed that human population density and distance to human population settlements are less important than temperature variables in our study. Furthermore, in urban areas, predictions based on temperatures should be treated with caution, as there may be microhabitats and refuges for mosquito populations where temperatures are higher, as demonstrated in Rome where this species overwinters as adults [39]. Curiously, although air temperatures in Trento during January 2009 went down to a minimum of −10°C and a mean of −5°C, tiger mosquitoes were still present in the following year, a fact that may support the hypothesis that the Italian populations are adapting to the cold.

Satellite based Land Surface Temperatures (LST) are equivalent to air temperatures measurements from meteorological stations [40], but with the advantage that they are already spatialised [35]. Therefore, they are excellent for regional scale forecasting. In this study, we observed that a mean January LST threshold of 0°C together with a mean annual LST threshold of 11°C provides an accurate explanation of the current distribution of *Ae. albopictus* in this area of Northeastern Italy (Fig. 3). We consider these LST thresholds to be equivalent to the widely used air temperature threshold of 0°C for JanT^mean^, which has been used in several studies for assessing the winter survival rate of eggs, and the threshold of 11°C for AnnT^mean^ for assessing adult survival, mosquito activity and relative abundance of *Ae. albopictus* [17], [18], [24], [26]. The two indicators fitted with each other, JanT^mean^ being the most important factor conditioning the survival of eggs in winter and hence the overwintering of the populations. A recent study confirmed that an air JanT^mean^ of 0°C is the limit for overwintering in the U.S.A. [41], corresponding to our identified 0°C LST JanT^mean^ threshold. In mountainous areas, temperature is a key factor in *Ae. albopictus* distribution and seasonal dynamics [6]. Altitude has also been used for defining the distribution limits of *Ae. albopictus* in Italy [2], [9]. However, our results show that altitude has limited predictive power since it only partially explains mosquito presence. Maps derived from satellite-based LST observations explain species presence in greater detail. The reason is that local temperature profiles are driven by factors such as orientation, shadow, insolation time, land cover, and slope, which are better captured by LST measurements. A simple elevation analysis cannot deliver this level of detail.

Mountainous regions such as the European Alps are considered particularly sensitive and vulnerable to meteorological and climate impacts caused by global warming. In fact, there has been a mean annual temperature increase in the Alps since 1890 of 1.1°C [42]. Brunetti et al. [43] also demonstrated a positive trend in mean temperature of about 1°C per century over all of Italy. Our simulation of the potential distribution was modeled on a future climate scenario based on previous work developed by Eccel et al. [38], [44]. For this, we used their data downscaled from the SRES (Special Report on Emissions Scenarios) A2 scenario which describes a highly heterogeneous world characterized by self-reliance, preservation of local identities [45], and a continuously increasing global population. Economic development is primarily regionally oriented and per capita economic growth and technological change are more fragmented and slower compared with other scenarios [45]. We decided to use this hypothetical A2 scenario for 2050 to represent the increase in the potential distribution areas of *Ae. albopictus* due to climate change. Experimental field work to be carried out over the next few years at the fringes of the suitable areas is currently being planned in order to assess whether expansion from suitable into unsuitable areas is already taking place. In future research, this model could be applied to a wider area. In addition, the phenomenon of cold-hardiness in temperate populations [15], which might increase the ability of *Ae. albopictus* eggs to survive in areas with low winter temperatures, should be investigated.

The results suggest that the tiger mosquito is spreading northwards. There is also a potential related risk of several mosquito-borne diseases spreading into new areas of Europe. However, we should distinguish between a possible spread of the species in Alpine scenarios and the risk of disease transmission, given that transmission of disease also depends on temperature [46]. We consider these predictions to be useful for developing plans to prevent further spread and establishment of this mosquito. Applied Europe-wide, these measures, consisting of an ‘early warning system’ (with the use of ovitraps) and subsequent vector control measures, could be highly effective. This study highlights the range limits for this species in mountainous areas and in central Europe, where winter temperatures and annual mean temperatures are the most important limiting factors in *Ae. albopictus* expansion. We have shown that the observed trend of increasing temperatures due to climate change could facilitate further invasion of the tiger mosquito into new areas.
